# Interpretation of uniocular and binocular trials of glaucoma medications: an observational case series

**DOI:** 10.1186/1471-2415-7-17

**Published:** 2007-10-05

**Authors:** Christopher T Leffler, Lina Amini

**Affiliations:** 1 Department of Ophthalmology, Virginia Commonwealth University, MCV Campus, MCV Box 980209, Richmond, VA 23298, USA; 2 UNC Department of Ophthalmology, Communications Center, 2nd Floor ACC Bldg CB 7720, Chapel Hill, NC 27599, USA

## Abstract

**Background:**

To predict the effectiveness of topical glaucoma medications based on initial uniocular and binocular treatment. To test a traditional hypothesis that effectiveness following a uniocular trial is associated with the change in IOP in the initially treated eye minus the change in the initially untreated eye. To determine whether uniocular or binocular treatment trials are superior.

**Methods:**

Based on a review of medical records, we identified 168 instances in 154 patients with bilateral primary open angle glaucoma of initial uniocular use of a topical glaucoma medication with well-documented intraocular pressure (IOP) readings at baseline (IOP_A_), during the trial (IOP_B_), and at follow-up (IOP_C_). Abstracted data included demographic data, IOP, and medication use. Predictors of the IOP following the trial (IOP_C_) in each eye were identified by multivariable linear regression. In 70 cases, the predictive ability of initial uniocular and binocular treatment could be directly compared.

**Results:**

In a multivariable analysis, the follow-up pressure in the initially treated eye (IOP_1C_) was directly correlated with treated eye IOP during initial uniocular use (IOP_1B_, p < 0.001). In a multivariable analysis, the follow-up pressure in the initially untreated eye (IOP_2C_) was directly correlated with its baseline IOP_2A _(p < 0.001), and also tended to be associated with treated IOP_1B _(p = 0.07). The multivariable regression coefficient (b) for the IOP change in the initially untreated eye was generally not close to the value of -1 expected by the classic teaching (for eye 1, b = 0.04, p = 0.35; for eye 2, b = 0.07, p = 0.50). In 70 cases, the uniocular and binocular trials predicted a similar fraction of the variance in follow-up IOP_1C _(r^2 ^= 0.56 and 0.57, respectively) and IOP_2C _(r^2 ^= 0.39 and 0.38, respectively).

**Conclusion:**

1) For uniocular trials, the IOP change in the untreated eye should not be subtracted from that in the treated eye. 2) Uniocular and binocular trials have similar predictive value when interpreted correctly. Either may be selected based on clinical circumstances.

## Background

Most authorities recommend a trial of topical glaucoma medications which are intended for long-term use in order to assess patient-specific effectiveness and tolerability. Classically, a uniocular drug trial has been recommended [1-3]. Drug effectiveness is felt to be related to the change in intraocular pressure (IOP) in the treated eye minus the change in IOP in the untreated eye [1,3-5]. Using the untreated eye as a control is thought to remove correlated variability due to diurnal fluctuation [1,4], operator technique, tonometer calibration, etc.

More recently, several objections to the uniocular drug trial have been raised. First, some glaucoma agents are known to have a significant crossover effect [6]. Second, spontaneous or diurnal IOP fluctuations in each eye appear to be poorly correlated [7-9]. Third, one study suggested that treatment effect in the first eye during a monocular trial is only weakly associated with treatment effect in the second eye [10]. These findings have led some investigators to suggest that the uniocular drug trial be abandoned [10,11] or modified [7,8,12], but these calls have not been universally accepted [13,14].

We performed a retrospective review of patients undergoing initiation of topical glaucoma therapy. The objectives were to determine: 1) how the information from uniocular and binocular trials can best be used to determine medication effectiveness, and 2) whether uniocular and binocular trials are equally successful at predicting medication effectiveness. We specifically sought to determine whether the classical teaching that effectiveness following a uniocular trial is related to the change in the treated eye minus the change in the untreated eye, where the untreated eye serves as a control.

## Methods

This study was in compliance with the Helsinki Declaration and was approved by the Office of Research Subjects Protection Institutional Review Board at Virginia Commonwealth University, Medical College of Virginia campus.

Medical records of glaucoma patients seen at the Medical College of Virginia were retrospectively reviewed. Inclusion criteria for eligible patients included: age over 18 years, diagnosis of bilateral primary open angle glaucoma, and initiation of topical glaucoma treatment initially in one eye with documented intraocular pressures by applanation in both eyes at each of three major time points. These time points included one or more baseline visits (time A), a uniocular treatment trial (time B), and during one or more follow-up visits (time C). The baseline visits occurred less than one year before the uniocular trial, and involved identical glaucoma treatments, with the exception of the uniocular trial treatment. The follow-up visits occurred less than one year following the uniocular trial, and involved identical glaucoma treatments, except that the trial treatment may have been used binocularly during follow-up.

Before this analysis was presented in 2003 at the Association for Research in Vision and Ophthalmology, it was standard practice by the residents and the majority of attendings at our institution to begin treatment with a uniocular trial. An obvious exception was when the physician felt that the IOP was bilaterally elevated to the degree that it would be unsafe to leave a control eye untreated.

When assessing drug effectiveness during a uniocular trial, it was standard clinical practice to subtract the IOP change in the untreated eye from the IOP change in the treated eye to account for diurnal and other correlated fluctuation. The standard practice and clinical judgment of the treating physician guided all treatment decisions, including whether to perform a uniocular trial, which eye to treat during the uniocular trial, and whether to treat binocularly on subsequent visits.

For several patients, a uniocular trial and follow-up visit data were obtained, but on subsequent visits, the physician decided to add an additional pressure-lowering agent with another uniocular trial. As long as satisfactory baseline and follow-up visit data were available, both trials were included in the data set. In general, the presence of concurrent glaucoma treatment was not an exclusion criterion. Patients on additional medications were included because advocates of uniocular or binocular trials have not stated that either type of trial only works for the first topical agent, but not for subsequent agents. It was confirmed that presence of concurrent topical glaucoma agents did not alter the study findings by analyzing separately trials in which the patients were treated and untreated at baseline.

In a subset of patients, uniocular and binocular trials could be directly compared in a secondary analysis because the uniocular treatment trial (visit B1) was followed by binocular use (visit B2), and then by additional follow-up visits with binocular medication use (time C).

Data collected from the medical records included demographic information, eye medications, and IOP. Predictors of the followup intraocular pressure in the initially treated eye (IOP_1C_), and the second eye (IOP_2C_), following a uniocular or binocular trial were evaluated by multivariable linear regression analysis. Potential predictors included the intraocular pressure in both eyes at baseline, and in eye 1 during initial treatment, and the change in IOP in the initially untreated eye. Regression residuals were plotted against the predicted values and the independent variables to verify an absence of correlations and to verify constancy of the variability of residuals [15]. Statistica software (version 7, Statsoft Inc., Tulsa OK) was used to determine the coefficient of determination (r^2^), the regression coefficient (b), normality of dependent variables by the Shapiro-Wilk test, and statistical significance values (p).

For the average follow-up IOP in the first treated eye, the degree of normality (Shapiro-Wilk p = 0.053) was improved by logarithmic (p = 0.54) transformation and improved even more by square root transformation (p = 0.66). Therefore, all analyses were repeated after square root transformation of the follow-up IOP values. Almost identical p and r^2 ^values were obtained with transformed analyses. Moreover, the relative weighting of terms was very similar. Logarithmic transformation of the IOP [16] also yielded nearly identical results. Because the conclusions were unchanged with transformation, and because the untransformed analyses provide the most direct comparison with classical teachings, the untransformed analyses are presented. However, the relevant p and r^2 ^values for the square root transformed analyses are presented to demonstrate the equivalence of the results.

## Results

One hundred sixty-eight eligible uniocular trials in 154 patients were identified and included in the analysis. A subset of 70 trials in 68 subjects involved uniocular use followed by binocular use for two or more visits.

Of the 168 uniocular trials, 44% were in males, and 78% were in black patients (Table 1). This finding simply reflects the demographic makeup of the university practice in an urban setting. The mean age was 62.7 years (Table 2). The medications used in the trial are listed in Table 1. The average intraocular pressure in the first eye fell from 20.2 mmHg at the final baseline visit (A), to 15.3 mmHg during the uniocular trial (visit B), and then increased slightly to 16.1 mmHg during follow-up (time C, Table 1). The average intraocular pressure in the second eye gradually decreased from 18.3 mmHg at the final baseline visit (A), to 17.4 mmHg during the uniocular trial (visit B), and then to 16.3 mmHg during follow-up (time C, Table 3).

**Table 1:** Characteristics of subjects (n = 154) and glaucoma drug trials (n = 168)

	Mean	(Standard deviation)	Number.	Per cent.
Male sex			68	44%
Black race (n = 142)			111	78%
Trial of:				
Latanoprost			86	51%
Timolol only			33	20%
Dorzolamide only			24	14%
Timolol/dorzolamide combination			1	1%
Brimonidine			17	10%
Bimatoprost			6	4%
Pilocarpine			1	1%
Age (years)	62.7	(11.8)		
Baseline IOP, eye 1* (mmHg)	20.2	(5.3)		
Baseline IOP, eye 2* (mmHg)	18.3	(5.2)		
Uniocular trial IOP, eye 1 (mmHg)	15.3	(4.0)		
Uniocular trial IOP, eye 2 (mmHg)	17.4	(5.6)		
Followup IOP, mean, eye 1 (mmHg)	16.1	(3.4)		
Followup IOP, mean, eye 2 (mmHg)	16.3	(4.5)		

* Eye 1 is the initially treated eye. Eye 2 is the other eye.

**Table 2:** Prediction of the average intraocular pressure in the initially treated eye (eye 1) following a uniocular trial by multivariable linear regression

Cases (n, r^2^, Intercept).	IOP, eye 1		IOP, eye 2
	
	Last baseline	During Trial	Change with trial.
	
	Time A	Time B	Time B – Time A.
	
	(b, p)	(b, p)	(b, **p**, p*)
All cases (168, 0.40, 6.9)	(0.27, <0.001)	(0.24, <0.001)	(0.04, **0.35**, 0.34)
Baseline untreated (99, 0.44, 6.4)	(0.24, 0.002)	(0.34, <0.001)	(0.07, **0.43**, 0.40)
Baseline treated (69, 0.36, 7.9)	(0.24, 0.002)	(0.20, 0.02)	(0.09, **0.10**, 0.09)
Latanoprost (86, 0.44, 5.6)	(0.37, <0.001)	(0.20, 0.09)	(0.03, **0.79**, 0.80)
Timolol only (33, 0.45, 8.2)	(0.22, 0.03)	(0.23, 0.06)	(0.05, **0.64**, 0.70)
Brimonidine (17, 0.51, 3.5)	(0.29, 0.06)	(0.43, 0.10)	(-0.27, **0.20**, 0.24)
Dorzolamide only (24, 0.61, 0.7)	(0.45, 0.004)	(0.48, 0.02)	(0.14, **0.04**, 0.04)

*p value for regression to predict square root of IOP, eye 1.

**Table 3:** Prediction of the average intraocular pressure during binocular treatment in the initially untreated eye (eye 2) by multivariable linear regression.

Cases (n, r^2^, Intercept).	IOP, eye 1		IOP, eye 2	
	
	Last baseline.	During Trial.	Baseline.	...change with uniocular trial.
	
	Time A.	Time B.	Time A.	Time B – A.
	
	(b, p)	(b, p)	(b, p)	(b, **p**, p*)
All cases (96, 0.49, 5.4)	(-0.002, 0.98)	(0.19, 0.07)	(0.42, <0.001)	(0.07, **0.50**, 0.53)
Baseline untreated (64, 0.47, 5.6)	(-0.01, 0.94)	(0.14, 0.30)	(0.44, 0.004)	(0.10, **0.54**, 0.55)
Baseline treated (32, 0.56, 4.4)	(0.03, 0.82)	(0.24, 0.17)	(0.41, 0.006)	(0.06, **0.72**, 0.80)
Latanoprost (56, 0.41, 5.4)	(0.05, 0.81)	(-0.004, 0.98)	(0.50, 0.03)	(0.25, **0.19**, 0.23)
Timolol only (19, 0.62, 6.0)	(0.02, 0.89)	(0.47, 0.04)	(0.14, 0.47)	(0.04, **0.86**, 0.72)
Dorzolamide only (8, 0.90, 2.1)	(0.38, 0.03)	(0.06, 0.84)	(0.78, 0.01)	(0.71, **0.03**, 0.04)
Brimonidine (10, 0.66, 4.6)	(0.17, 0.58)	(0.04, 0.92)	(0.39, 0.23)	(0.04, **0.90**, 0.86)

*p value for regression to predict square root of IOP, eye 2.

### Uniocular trial prediction of first eye IOP

The significant independent predictors of follow-up IOP_1C _in the first-treated eye by multivariable analysis were the most recent baseline IOP_1A _and the IOP_1B _during the trial (p < 0.001, r^2 ^= 0.40, Table 2).

On the whole, the change in second eye IOP during the trial (IOP_2B _– IOP_2A_) was not a significant predictor of follow-up IOP_1C _(p = 0.35, Table 2). Only for the 24 trials of dorzolamide was the second eye term significantly positive (b = 0.14, p = 0.04, Table 2). For brimonidine, the coefficient tended to be negative (b = -0.27, p = 0.20), but the magnitude was much smaller than the value of -1 used in traditional clinical practice.

### Uniocular trial prediction of second eye IOP

For the initially untreated eye follow-up IOP_2C_, the baseline IOP_2A _was an independent predictor (p < 0.001) and the treated IOP_1B _during the uniocular trial was nearly significant (p = 0.07, Table 3). The regression coefficient for the second-eye IOP change during the trial (IOP_2B _– IOP_2A_) was not close to the value of -1 expected in the classical teaching (b = 0.07, p = 0.50). In fact, for dorzolamide, the coefficient was significantly positive (b = 0.71, p = 0.03, Table 3).

### Comparison of uniocular and binocular trials

In 70 cases, a uniocular trial was followed by at least two visits with binocular use. The first visit with binocular use may be regarded as a binocular trial, while IOP on subsequent follow-up visits may serve as the dependent analytic variable. The independent predictors of the average followup pressure in the initially treated eye were the eye 1 IOP at baseline and during the uniocular or binocular trial (Table 4). The models based on uniocular and binocular use of the medication predicted similar fractions of the variance (r^2 ^= 0.56 and 0.57, respectively, Table 4). The change in intraocular pressure in the contralateral eye (2) during the binocular trial was not a significant independent predictor (p = 0.16, Table 4).

**Table 4:** Predictors of the average intraocular pressure in the initially treated eye (eye 1) following a uniocular trial and subsequent binocular trial of medication by multivariable linear regression.

Cases...trial type (r^2^, r^2^*, Intercept).	IOP, eye 1		IOP, eye 2	
	
	Last baseline.	During Trial.	Baseline.	Change with trial.
	
	Time A	Time B	Time A	Time B – A.
	
	(b, p)	(b, p)	(b, p)	(b, p)
All cases (n = 70). Trial type...				
...uniocular (0.56, 0.55, 4.5)	(0.38, <0.001)	(0.29, 0.02)	(-0.005, 0.96)	(-0.10, 0.41)
...binocular (0.57, 0.57, 3.6)	(0.45, <0.001)	(0.07, 0.70)	(0.20, 0.16)	(0.27, 0.16)
Baseline untreated (n = 48). Trial type...				
...uniocular (0.55, 0.54, 4.7)	(0.36, 0.02)	(0.26, 0.10)	(0.02, 0.89)	(-0.10, 0.56)
...binocular (0.56, 0.57, 3.4)	(0.39, 0.01)	(0.14, 0.51)	(0.20, 0.35)	(0.22, 0.39)
Baseline treated (n = 22). Trial type...				
...uniocular (0.60, 0.59, 3.4)	(0.40, 0.006)	(0.34, 0.11)	(0.02, 0.92)	(-0.02, 0.91)
...binocular (0.60, 0.59, 4.3)	(0.56, 0.003)	(-0.17, 0.61)	(0.30, 0.19)	(0.44, 0.19)

* r^2 ^value for regression to predict square root of IOP, eye 1.

The ability to predict the second eye IOP also was similar between uniocular (r^2 ^= 0.39) and binocular trials (r^2 ^= 0.38, Table 5). In these analyses, only the baseline second eye IOP_2A _was a significant predictor of second eye final IOP (p < 0.05, Table 5).

**Table 5:** Predictors of the average intraocular pressure during binocular use in the subsequently treated eye (eye 2) following a uniocular trial and subsequent binocular trial of medication by multivariable linear regression.

Cases...trial type (r^2^, r^2^*, Intercept).	IOP, eye 1		IOP, eye 2	
	
	Last baseline.	During Trial.	Baseline.	Change with trial.
	
	Time A	Time B	Time A	Time B – A.
	
	(b, p)	(b, p)	(b, p)	(b, p)
All cases (n = 70). Trial type...				
...uniocular (0.39, 0.39, 5.4)	(0.11, 0.27)	(0.16, 0.22)	(0.33, 0.008)	(0.13, 0.37)
...binocular (0.38, 0.39, 6.0)	(0.17, 0.14)	(-0.10, 0.60)	(0.51, 0.003)	(0.35, 0.12)
Baseline untreated (n = 48). Trial type...				
...uniocular (0.40, 0.42, 4.8)	(0.04, 0.80)	(0.15, 0.40)	(0.44, 0.03)	(0.12, 0.57)
...binocular (0.40, 0.43, 5.0)	(0.07, 0.70)	(-0.09, 0.72)	(0.67, 0.008)	(0.35, 0.24)
Baseline treated (n = 22). Trial type...				
...uniocular (0.37, 0.36, 6.8)	(0.14, 0.31)	(0.12, 0.60)	(0.28, 0.10)	(0.19, 0.43)
...binocular (0.37, 0.36, 7.9)	(0.24, 0.17)	(-0.19, 0.58)	(0.42, 0.09)	(0.38, 0.28)

* r^2 ^value for regression to predict square root of IOP, eye 2.

## Discussion

There is currently debate in the ophthalmology community about whether treatment with topical glaucoma agents should be initiated in one eye or in both eyes [7,8,10,12-14]. Classically, it was felt that a more reliable estimate of drug effect could be obtained with a uniocular trial. The IOP change in the untreated eye was subtracted from the change in the treated eye, to account for correlated variation between the eyes.

This retrospective review of initiation of topical glaucoma therapy has demonstrated that: 1) The traditional teaching that for uniocular trials the IOP change in the untreated eye should be subtracted from that of the treated eye to control for diurnal and other correlated fluctuations does not universally apply. 2) Uniocular and binocular trials have similar predictive value when interpreted correctly, and either may be selected based on clinical circumstances.

The reference standard for determining drug effectiveness in an individual patient is to randomly alternate administration of the drug or a placebo in a double-masked fashion over multiple visits at which the outcome variable is assessed (an "n-of-1 trial") [17,18]. With respect to glaucoma, drug or placebo drops would be randomly administered and IOP would be recorded. This technique is not currently standard in the treatment of glaucoma. In this study, we assumed that the average IOP on treatment, following any uniocular or binocular medication trials, minus the pre-treatment average IOP, is the best available estimate of the true drug effect [11]. Because the pre-treatment average IOP is known at the time of the trial, the only unknown which must be estimated to know the drug effect is therefore average follow-up IOP. This follow-up IOP was therefore the primary dependent variable in the main regression analyses.

Some authors have suggested that the uniocular drug trial must be abandoned because of crossover drug effect [6], because the contralateral eye IOP change during a single visit may not be used as a control due to uncorrelated fluctuations (unrelated to drug effect) [7,9], or because the drug effect itself may be independent between the two eyes [10]. Our study addressed these questions regarding the validity of the uniocular drug trial as traditionally proposed.

The major finding at odds with standard clinical teaching is that the IOP change in the untreated eye during a uniocular trial should not be subtracted when assessing drug effectiveness. An example will help to illustrate the importance of this finding. A typical patient has a baseline IOP of 25 mmHg in both eyes, treatment is initiated in one eye, and the follow-up IOP is 20 mmHg in both eyes. The standard clinical teaching is that the drug is ineffective in this patient because the bilateral IOP change relates to diurnal or other correlated fluctuation. However, this teaching involves several assumptions: the spontaneous IOP variation is correlated between both eyes and drug crossover effect is minimal. An additional assumption is that the patient, day, and eye are selected for initiation of treatment at random, so that there is no regression to the mean. According to the classic teaching, the regression coefficient for the IOP change in the untreated eye is minus one in these circumstances. When the change in the untreated eye is subtracted from the change in the treated eye, it appears that the drug had no effect.

Alternate interpretations can be made if these assumptions do not apply. For instance, a bilateral drop in IOP from 25 to 20 mmHg with uniocular treatment may in theory also be interpreted as the result of an effective drug with a substantial crossover effect. If drug crossover is so substantial that both eyes essentially receive an equal effect even with unilateral treatment (a theoretical situation which does not apply to currently available topical agents), then the drug effect can be estimated by the average IOP change. As the average change is simply one half the change in the treated eye plus one half the change in the untreated eye, the regression coefficient for the IOP change in the untreated eye would tend to plus one half with a complete crossover effect.

Another factor which may affect the interpretation is regression to the mean, which occurs in clinical practice (and even in many randomized controlled trials) because glaucoma treatments are only initiated when patients have an IOP above the target pressure. As the IOP may be above the target pressure only due to random fluctuation, as opposed to a true change in the underlying disease process, subsequent visits may see a drop in IOP simply due to the statistical phenomenon known as regression to the mean. This phenomenon applies to both uniocular and binocular trials in clinical practice. In uniocular trials it occurs not only because the average IOP may be high on a particular visit, but also because most clinicians (and randomized trial protocols [19]) choose the eye with the higher IOP for initial treatment. Regression to the mean likely explains much of the 5% drop in IOP seen with placebo treatment in randomized trials [20]. It is possible to reduce regression to the mean in a research setting by selecting random patients, asking them to come to the clinic on random days, and initiating treatment in one eye or in both eyes at random, regardless of whether the patient appears to need additional treatment. Such a protocol may have research value but involves initiating therapy in patients already below their target pressure. Because physicians only treat patients who appear to need treatment, regression to the mean is a fact of life with which clinicians must contend when trying to understand clinical observations. The statistical coefficients derived in this study allow clinicians to make predictions which take into account regression to the mean.

In most analyses, the IOP change in the untreated eye during a uniocular trial had minimal predictive value and the regression coefficient was close to zero. For dorzolamide, the change in IOP in the untreated eye had significant predictive value, but in the direction opposite from the standard clinical teaching. Although a more positive coefficient might suggest the importance of crossover effects with dorzolamide [21], the number of dorzolamide trials was small and this finding may be due to chance.

Our data indicate that the uniocular drug trial need not be abandoned in all cases. However, the interpretation of the uniocular trial must be modified. After a uniocular trial, regression equations may be used to predict the IOP in the first eye (Tables 2) and in the second eye (Table 3). Likewise, after a binocular trial, regression equations may be used to predict the IOP in the first (Table 4) or second eye (Table 5). When interpreted correctly, both uniocular and binocular trials had similar success in predicting the future IOP in the first or second eye.

Whether a uniocular or binocular trial is performed depends on a variety of circumstances. From the standpoint of safety, the uniocular trial has advantages because the patient presumably will receive half the dose systemically, and because any local allergy or intolerance will be more easily diagnosed by its uniocular presence. Drug tolerability was an important aspect of initial calls for uniocular trials [2]. On the other hand, the binocular drug trial succeeds in providing benefit to both eyes more quickly and may spare the patient an additional visit. Moreover, a binocular trial is less confusing because it avoids subsequent regimen changes, and because the patient need not remember which drug is initially taken uniocularly and which eye is treated.

This study had a number of limitations. First, several subjects contributed more than one trial with different types of medication even though the results of two trials of different medications in one individual might not be completely independent. This objection is largely addressed by the subset of trials comparing uniocular and binocular trials because only two of the 68 subjects contributed more than one medication trial. Finally, as with previous studies [9-11], the data were retrospective and patient compliance may have been imperfect. On the other hand, the data reflect real-world observations made in an urban university glaucoma practice, and may apply to similar practices.

One problem highlighted by our data is that a single-visit uniocular or binocular drug trial still leaves a great deal of unexplained variation in follow-up IOP. Even combining the information from two visits (a uniocular and binocular trial) had minimal added benefit in terms of the ability to predict IOP (data not shown). Some authors have hypothesized that plotting the diurnal variation in individual patients might make estimation of drug effect with uniocular trials more accurate [7,8,12,22]. Others have advocated multiple baseline and follow-up visits in the context of binocular drug trials [11]. Undoubtedly, additional visits will permit better predictions for both uniocular and binocular drug trials, but a quantitative understanding of the improvement is lacking. Additional studies will be needed to test specific protocols. Given the difficulty of assessing patient-specific therapeutic effect, in the future appropriate medication selection may also be based in part on genetic testing [23].

## Conclusion

To briefly summarize, our study showed that the change in IOP in the untreated eye during a uniocular trial does not generally provide significant predictive information. Either uniocular or binocular trials may be performed in appropriate clinical circumstances.

## Competing interests

The author(s) declare that they have no competing interests.

## Authors' contributions

CL conceived of and designed the study, performed data analysis and interpretation, and drafted the manuscript.

LA participated in design of the study, obtained data from medical records, and helped with data interpretation and drafting the manuscript.

Both authors read and approved the final manuscript.

## Pre-publication history

The pre-publication history for this paper can be accessed here:


